# Outcomes of Percutaneous Coronary Intervention in Patients with Spontaneous Coronary Artery Dissection

**DOI:** 10.1155/2021/6686230

**Published:** 2021-05-15

**Authors:** Saber Hassan, Rohit Samuel, Andrew Starovoytov, Carolyn Lee, Eve Aymong, Jacqueline Saw

**Affiliations:** Division of Cardiology, Vancouver General Hospital, University of British Columbia, Vancouver, Canada

## Abstract

**Objectives:**

To compare outcomes of percutaneous coronary intervention (PCI) in spontaneous coronary artery dissection (SCAD) patients versus conservative therapy.

**Background:**

SCAD is an important cause of myocardial infarction (MI) in young-to-middle-aged women. Percutaneous coronary intervention (PCI) is often pursued, but outcomes compared to conservative therapy are unclear.

**Methods:**

403 nonatherosclerotic SCAD patients were enrolled between 2011 and 2017 and prospectively followed up in our Vancouver General Hospital registries. Detailed baseline, hospital, PCI, and outcomes were recorded. We explored the outcomes of SCAD patients who underwent PCI during their initial presentation.

**Results:**

PCI was performed in 75 patients, the average age was 48.9 ± 10.1 yrs, and 94.7% were women. All presented with MI; 50.7% STEMI, 49.3% NSTEMI, and 13.3% had VT/VF. PCI was successful in 34.7%, partially successful in 37.3%, and unsuccessful in 28.0%. Stents were deployed in 73.3%, 16.0% had balloon angioplasty alone, 10.7% had wiring attempts only, and 5.3% required bailout surgery. Major adverse cardiovascular event rates (MACE) were significantly higher with the PCI group in hospital (29.3% versus 2.8%, *p* < 0.001), and at median follow-up of 3.7 yrs (58.7% versus 22.6% (*p* < 0.001) compared to the non-PCI group.

**Conclusion:**

PCI in SCAD patients was associated with high failure rate and MACE in hospital and at long-term follow-up. These findings support the recommendation of conservative therapy as first-line management unless high-risk features are present.

## 1. Introduction

Spontaneous Coronary Artery Dissection (SCAD) is defined as a spontaneous, nontraumatic, and noniatrogenic dissection of the coronary arterial wall, not related to atherosclerosis [1]. SCAD is characterized by the development of intramural hematoma with or without intimal disruption, leading to variable degrees of luminal narrowing causing ischemic myocardial injury. The origin of the intramural hematoma within the arterial wall could be from intimal tear leading to dissection or from bleeding due to ruptured vasa vasorum in the absence of an intimal tear [2, 3].

The true prevalence of SCAD is unknown, mostly because it remains an underdiagnosed condition and underreported cause of acute coronary syndrome (ACS). Recent studies showed that SCAD was the cause of 0.1–4% of all ACS cases [1, 4]. It is more prevalent in young-to-middle-aged women. It was reported to cause 24–35% of ACS among women under fifty years of age [5–7] and was the most common cause of ACS during pregnancy (43%) [8].

The optimal treatment strategy for SCAD remains controversial, although conservative therapy is generally the preferred strategy, particularly for stable patients with no further evidence of ischemia. Revascularization is typically pursued for unstable patients, such as those with ongoing ischemia, hemodynamic instability, or left main involvement [9, 10]. Percutaneous coronary intervention (PCI) is the preferred revascularization modality, and coronary artery bypass grafting (CABG) is relegated as a bailout strategy for failed PCI or for patients with left main dissection. One of the key rationales for conservative management is derived from the observation that spontaneous angiographic healing occurs in the vast majority of cases after about a month [11]. Furthermore, small retrospective series had shown that PCI of SCAD lesions can be challenging and associated with poor outcomes [1].

There has been no randomized controlled trial performed to date comparing conservative therapy to PCI in patients presenting with acute SCAD, and published observational series assessing PCI outcomes with SCAD were small and lack long-term follow-up. Therefore, the objective of our study is to assess the acute and long-term outcomes in a relatively large cohort of SCAD patients who underwent PCI and compare the outcomes to conservatively managed SCAD patients.

## 2. Methods

We retrospectively analyzed our cohort of nonatherosclerotic SCAD patients who were enrolled in our Vancouver General Hospital SCAD registries and prospectively followed up at the Vancouver SCAD Clinic. We included patients who underwent PCI and compared their outcomes to patients treated conservatively. All patients provided informed consent for our SCAD registries approved by the University of British Columbia Research Ethics Board. Patients were interviewed and completed detailed questionnaires on potential predisposing and precipitating stressors, gynecologic history, clinical symptoms, and family history. Detailed baseline demographics, medical history, clinical presentation, laboratory results, angiographic findings, and PCI procedural details were recorded. In-hospital and long-term cardiovascular events were collected. Screening for extracoronary fibromuscular dysplasia (FMD) in 3 arterial territories (renal, iliac, and cerebrovascular) was performed with catheter angiography or CT/MR angiography. All patients were followed up at least annually at the SCAD clinic or by telephone follow-up.

All coronary angiograms were reviewed by two experienced cardiologists for SCAD diagnosis and angiographic classification as previously described [12–14]. Type 1 angiographic SCAD describes the classic appearance of contrast dye staining of arterial wall with multiple radiolucent lumens. Type 2 angiographic SCAD describes diffuse smooth narrowing that can vary in severity; variant 2A has normal arterial segments proximal and distal to the dissection, whereas variant 2B has dissection that extends to the distal tip of the artery. Type 3 angiographic SCAD describes focal or tubular stenosis that mimics atherosclerosis, and usually requires optical coherence tomography (OCT) or intravascular ultrasound (IVUS) to prove intramural hematoma or double lumen. The coronary artery segment dissected was defined by the Bypass Angioplasty Revascularization Investigation Classification [2, 15]. Lesion length and stenosis severity were measured by quantitative coronary analysis (QCA).

**Figure 1:**
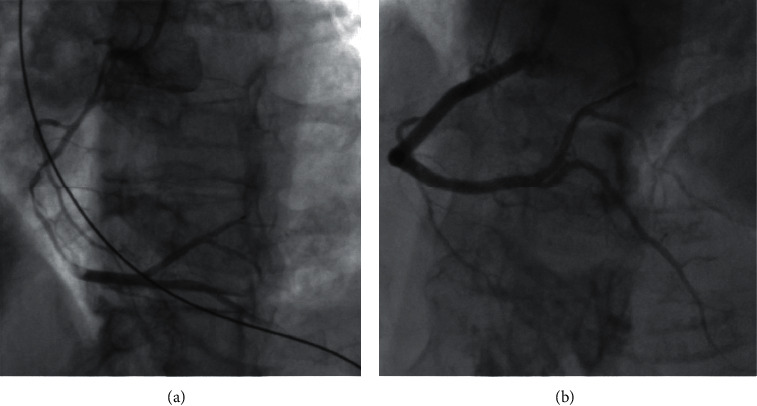
(a) Type 2A SCAD of the proximal to distal RCA. (b) Successful stenting of the proximal to distal RCA with final TIMI 3 flow.

**Figure 2:**
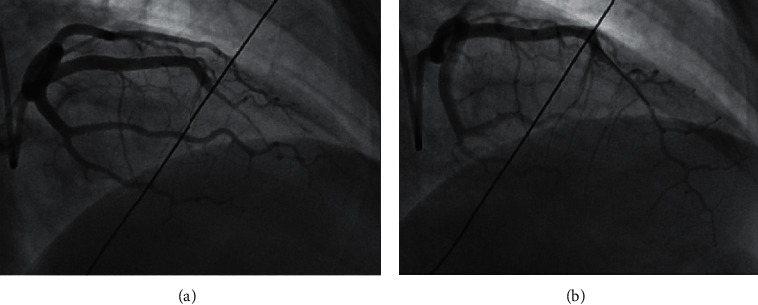
(a) Type 2B SCAD of the mid to apical LAD. (b) Partial successful PCI of the mid to apical LAD with final TIMI 3 flow.

**Figure 3:**
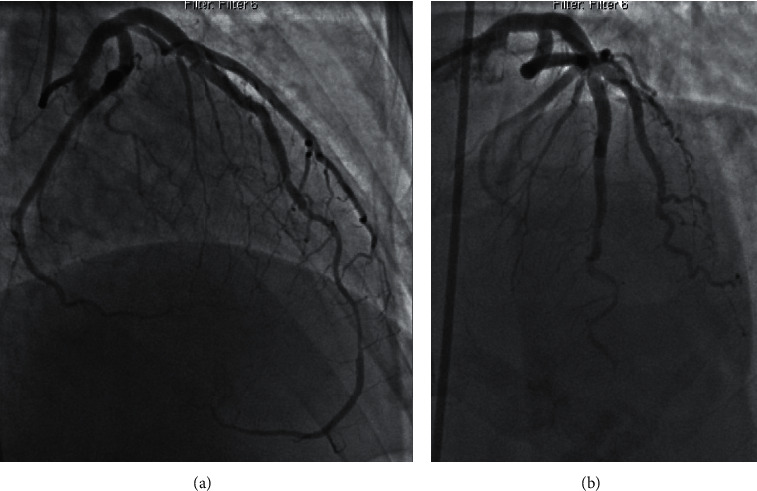
(a) Type 2A SCAD of the mid to distal LAD. (b) Unsuccessful PCI with occluded distal LAD and final TIMI 0 flow.

PCI outcomes were defined as follows: (a) successful PCI was defined as angioplasty or stenting of the dissection with TIMI 3 flow and no residual dissection (Figure 1); (b) partially successful PCI was defined as angioplasty or stenting with residual dissection or stenosis ≤50% of lumen diameter, and with final TIMI 3 or improved flow (Figure 2); and (c) unsuccessful PCI was defined as angioplasty or stenting with residual dissection or stenosis >50% of lumen diameter or worsened TIMI flow compared to baseline or extension of dissection requiring bailout CABG (Figure 3). Spontaneous angiographic healing at follow-up angiography was defined as angiographic resolution of the coronary dissection with residual stenosis <50% and no further evidence of multiple lumen or contrast wall staining.

In-hospital major adverse events (MAEs) were defined as a composite of all-cause mortality, stroke, reinfarction, cardiogenic shock, congestive heart failure, severe ventricular arrhythmia (requiring defibrillation or antiarrhythmic agents), repeat revascularization (or unplanned revascularization), and cardiac transplantation. Long-term major adverse cardiovascular events (MACEs) included a composite of all-cause mortality, stroke, recurrent MI (including recurrent SCAD), congestive heart failure, and revascularization. SCAD extension was defined as proximal or distal extension of the original SCAD lesion. Recurrent SCAD was defined as de novo recurrent spontaneous dissection with new recurrent MI symptoms and cardiac biomarkers elevation, which did not involve extension of dissection of the original SCAD lesion.

### 2.1. Statistical Analysis

Descriptive statistics were used to summarize baseline characteristics. Continuous variables were reported as mean ± SD or median and interquartile range. Categorical variables were reported as frequency and percentage. Categorical data were compared with the chi-square or Fisher's exact tests. Continuous data were compared using Student's *t*-test or Mann–Whitney test. Two-sided *p* values of <0.05 were considered significant. The log-rank test was performed to compare groups in survival analysis. Statistical analyses were performed with the SPSS software (IBM SPSS version 23, New York).

## 3. Results

Four hundred and three SCAD patients enrolled between 2011 and 2017 were included in this analysis; 75 (18.6%) underwent PCI of the SCAD-affected artery, and 328 (81.4%) were treated conservatively during their initial SCAD hospitalization. Baseline characteristics, risk factors, predisposing/precipitating factors, and hospital presentation of the patients are shown in Table 1. Patients in both groups were predominantly female and Caucasian. The mean age was lower in the PCI group (48.9 ± 10.1 yrs) compared to the non-PCI group (53.1 ± 9.6 yrs), *p*=0.001. The PCI group also had a lower prevalence of hypertension (25.3% versus 38.7%, *p*=0.033) and FMD (46.7% versus 64.0%, *p*=0.008) and were less likely to be postmenopausal (50.7% versus 67.7%, *p*=0.009). No patients were lost to follow-up in the trial period.

All patients presented with ACS and troponin elevation. More patients in the PCI group presented with ST-elevation MI (STEMI), 50.7% versus 19.8%, *p* < 0.001. Troponin-I level was higher in the PCI group (16.0 versus 6.1 ng/ml, *p*=0.001). High-sensitivity troponin-T level was numerically higher, but not statistically significant. Left ventricular ejection fraction (LVEF) was significantly lower in the PCI group (50.0% versus 58.0%, *p* < 0.001), with more PCI patients having LVEF<50% (40.3% versus 18.1%, *p*=0.001).

Angiographic results are shown in Table 2. The left anterior descending artery was the most commonly involved dissected artery (49.1%), followed by the circumflex artery (32.5%) and the right coronary artery (26.1%), which was not statistically different between groups. Dissection of the left main coronary artery occurred in only four patients (1.0%), and all underwent PCI (*p*=0.001). Multivessel noncontiguous SCAD occurred in 9.9% of patients. The PCI group had longer lesion lengths (median length 49 versus 39.3 mm, *p*=0.038), more severe stenosis (median stenosis 90.4% versus 77.6%, *p*=0.001), and greater proportion with severe stenosis 90–99% (57.4% versus 34.3%, *p*=0.001) and involved larger reference arteries (median diameter 2.8 versus 2.2 mm, *p* < 0.001). A greater proportion of patients in the PCI group had dissection involving the left main or proximal arteries (25.3% versus 4.6%, *p* < 0.001), TIMI 0 or 1 flow (49.3% versus 28.7%, *p*=0.001), and type 1 angiographic SCAD (37.3% versus 25.6%, *p*=0.046).

Of the 75 SCAD patients who underwent PCI, 60 had PCI as their first treatment strategy (80.0%), 11 had PCI after failed initial medical treatment (14.6%), and 4 had PCI after thrombolysis (5.3%). The rationale for PCI is described in Table 3. PCI was deemed successful in 34.7% (26/75), partially successful in 37.3% (28/75), and unsuccessful in 28.0% (21/75) (Table 4). Of the PCI procedures, the majority (73.3%) had stent implantation (5/55 were unsuccessful), angioplasty alone was performed in 16.0% (8/12 cases were unsuccessful), and wiring alone was attempted in 10.7% (8/8 were unsuccessful). Cutting balloon was used in only one case. The mean number of stents implanted was 2.6 ± 1.8, and more than three stents were used in 11 cases (15%). Of all PCI cases, propagation of SCAD occurred in 33 cases (44.0%), and residual dissection was observed in 44 (58.7%). Final TIMI 3 flow was observed in 54 patients (72.0%), and improved TIMI flow with PCI occurred in 47 cases (62.7%). Four patients required emergency bailout CABG (5.3%).

**Figure 4:**
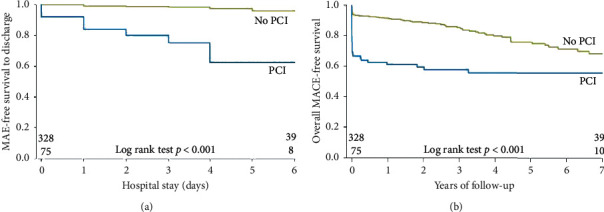
Kaplan–Meier event-free survival curves for PCI versus non-PCI group: (a) in-hospital major adverse events and (b) overall major adverse cardiovascular events.

Clinical events are shown in Table 5. The median length of hospital stay is 3 days [3, 4]. Patients in the PCI group had a higher incidence of in-hospital MI (20.0% versus 1.2%, *p* < 0.001), repeat revascularization (18.7% versus 0.9%, *p* < 0.001), stroke (4.0% versus 0.6%, *p*=0.047), and overall MAE (29.3% versus 2.8%, *p* < 0.001) compared to the non-PCI group (Figure 4(a)). The median long-term follow-up was 3.7 (2.1–5.9) years. After discharge, repeat revascularization remained higher with the PCI group (14.7% versus 3.0%, *p* < 0.001), and admission for unstable angina was also higher (13.3% versus 5.8%, *p*=0.043). The overall MACE at long-term follow-up was significantly higher in the PCI group (58.7% versus 22.6%, *p* < 0.001) (Figure 4(b)). Among conservatively managed patients, repeat angiography was performed in 132 patients, and angiographic healing was observed in 85.6%.

## 4. Discussion

We performed a retrospective analysis comparing the clinical outcomes of those who underwent PCI at the index SCAD event compared to those treated conservatively. Patients who underwent PCI had higher-risk baseline characteristics, including more STEMI presentations, worse LVEF, higher troponin elevation, longer lesion length, greater stenosis severity, larger-diameter arteries dissected, and more left main and proximal artery dissections. PCI procedures were successful or partially successful in 72% of cases and unsuccessful in 28%. The majority (73.3%) were treated with stent placement. Both in-hospital MAE and overall MACE at long-term follow-up were higher in the PCI group, primarily driven by rates of repeat revascularization and overall MI.

Acute management of SCAD remains challenging mainly due the lack of randomized trials and the fact that this disease is still underrecognized and underreported. Conservative therapy is recommended as first-line management based on expert consensus from observational and retrospective studies [9, 10]. However, SCAD patients with high-risk characteristics such as ongoing ischemia, hemodynamic instability, or left main dissection often require urgent revascularization to relieve ischemia and provide myocardial salvage. Unfortunately, PCI of SCAD-affected arteries can often be challenging and carries a high risk of failure and suboptimal outcomes [1]. Reported success rates of PCI with SCAD lesions ranged from 47%–91% in small retrospective series [1, 12, 13, 16, 18, 19]. SCAD-affected arteries are more susceptible to iatrogenic catheter-induced dissection [20] and propagation of the dissection induced by wire manipulation, angioplasty, or stenting, which can lead to antegrade or retrograde extension of dissections during PCI. SCAD also usually involves long segments of arteries requiring long stents, which can increase the risk of stent restenosis and thrombosis. Furthermore, undersizing of the stents in the setting of intramural hematoma may result in malapposition of the struts after resorption of the hematoma, with increased risk of late in-stent thrombosis [17]. Therefore, PCI for SCAD lesions should be reserved for patients with clinical high-risk features, and a suggested algorithm on the PCI strategy with SCAD was recently described [21].

Previous retrospective studies have reported on the suboptimal outcomes with SCAD PCI. Our current series differs in being one of the largest series and with details on PCI rationale and strategies and comparative long-term outcomes to a large cohort of patients managed conservatively. In our series, only a small and selected proportion of patients (18.6%) underwent PCI in the overall cohort, with the most common rationale for PCI being ongoing ischemia/symptoms, hemodynamic instability, severe stenosis, proximal artery involvement, and TIMI 0 or 1 flow. In-hospital MAE was higher in the PCI group, with higher recurrent MI, repeat revascularization, and stroke. After discharge, repeat revascularization and admission for unstable angina remained higher with the PCI group. In the Mayo Clinic series of 187 SCAD patients, almost half of their patients (87/187, 46.5%) were treated with PCI, suggesting a much lower threshold for intervention in their series (rationale for PCI not provided). Their procedural success rate was only 47%, which is lower than our reported rate, and this may be related to differences in definition of PCI success used. As well, the PCI strategy (i.e., proportion receiving stents or angioplasty alone) in this study was not reported. A higher proportion (13%) of their patients required bailout CABG. They found no difference in the 5-year rates of target vessel revascularization and recurrent SCAD in PCI versus conservative therapy groups (30% versus 19%, *p*=0.06; 23% versus 31%, *p*=0.7, respectively) [13]. In contrast, we found higher rates of repeat revascularization both in hospital and following discharge in the PCI group. It is difficult to compare the differences in repeat revascularization in these 2 studies, since there may be differences in PCI strategies and subjective indications for reintervention.

In the series by Lettieri of 134 SCAD patients, 51 underwent PCI (41.8%) and 5 (3.7%) underwent CABG. Successful PCI was achieved in 72.5%, and patients treated conservatively had lower in-hospital MACE compared with those treated with revascularization (3.8% versus 16.1%, *p*=0.028). Unsuccessful PCI was defined as a lack of improvement or worsened TIMI flow compared with baseline before PCI or extension of dissection. Three patients (5.8%) required bailout CABG after failed PCI. There were 5 repeat revascularizations in the PCI group [16]. In a meta-analysis of 11 studies published by Martins, among 631 SCAD patients in these nonrandomized studies, 253 were treated with PCI or CABG as the initial strategy. They found no difference in mortality, MI, or SCAD recurrence with revascularization versus conservative therapy. However, revascularization was associated with an estimated additional risk of target vessel revascularization of 6.3% [22]. These findings are concordant with our study.

In our study, the majority of patients who underwent PCI were treated with stents (73.3%), and angioplasty alone was performed in 16.0%. In 10.7%, wiring was unsuccessful. The optimal approach with PCI in SCAD lesions is unclear at this point, with several strategies that are feasible including balloon angioplasty alone, cutting balloon fenestration, sequential stenting, stenting edges first before middle, and use of bioabsorbable stents [21]. The selected PCI approach should be individualized and may be guided according to the anatomic appearance and extent of dissection. Nevertheless, our study supports the current recommendations that conservative therapy should be first-line, unless patients have high-risk features. Furthermore, studies that assessed repeat angiography for conservatively managed SCAD patients also showed that spontaneous healing occurs in the vast majority of cases (95% after 30 days) [11]. In the current series, ∼86% of conservatively managed patients had spontaneous healing on repeat angiography. The American Heart Association SCAD Scientific Statement recommended conservative management for clinically stable patients with no high-risk anatomy; PCI or CABG should be considered for patients with active or ongoing ischemia or hemodynamic instability. In stable patients with left main or severe proximal 2-vessel dissection, CABG or conservative management may be considered [9]. In the European Society of Cardiology SCAD position paper, it was highlighted that revascularization was associated with an increased risk of complications, and a conservative approach was recommended in the absence of ongoing ischemia [10].

## 5. Limitations

Our study is retrospective and observational and, therefore, subject to bias of patient selection for PCI versus conservative therapy. Selection of a treatment strategy was at the discretion of the operator, and provision of the rationale for PCI indicated that more high-risk patients were selected for PCI. Indeed, patients who underwent PCI had larger MIs, worse LVEF, and more ominous geographic SCAD anatomy. Thus, the worse outcomes observed with PCI may be related to these differences in baseline demographics, in addition to the high procedural failure. Interestingly, although overall MACE was higher with the PCI group, this was primarily driven by repeat revascularization and in hospital MI, with no significant difference in long-term mortality, recurrent MI, or recurrent SCAD. This suggests that the dominant consequence of PCI in SCAD arteries is related to mechanical interventional issues, as opposed to systemic effects, and does not affect future risk of SCAD.

## 6. Conclusions

In our large SCAD series, patients who underwent PCI had higher baseline and angiographic risk characteristics. PCI was associated with low procedural success and higher in-hospital complications of recurrent MI, repeat revascularization, and stroke, and also long-term risk of repeat revascularization, compared to conservative therapy. Our study supports the current societal expert recommendations of conservative therapy as first-line treatment for SCAD patients. Ideally, a randomized study comparing PCI to conservative therapy should be performed; however, the logistics of treating high-risk SCAD patients (e.g., left main dissection, ongoing ischemia) conservatively or treating low-risk SCAD patients with PCI (where most heal spontaneously) are fraught with ethical challenges and are against current practice recommendations. Therefore, although our study is observational and retrospective, this is currently the best-available evidence to evaluate revascularization indications and strategies with SCAD. Further studies are required to assess contemporary mechanical strategies to improve SCAD PCI outcomes.

## Figures and Tables

**Table 1:** Baseline and hospital characteristics.

Mean ± SD, median (Q1, Q3), or *n* (%)	PCI (*n* = 75)	No PCI (*n* = 328)	*P* value
Age	48.9 ± 10.1	53.1 ± 9.6	0.001
Female	71 (94.7)	297 (90.5)	0.363
BMI	22.7(20.7, 26.4)	24.7 (21.5, 28.8)	0.016
*Race*			0.890
Caucasian	63 (84.0)	271 (82.6)	
East Asian	8 (10.7)	34 (10.4)	
South Asian	3 (4.0)	16 (4.9)	
African Canadian	1 (1.3)	2 (0.6)	
First Nation	0 (0)	3 (0.9)	
Family history of CAD	23 (30.7)	114 (34.8)	0.589
HTN	19 (25.3)	127 (38.7)	0.033
Hyperlipidemia	20 (26.7)	81 (24.7)	0.768
DM	3 (4.0)	13 (4.0)	>0.9
Current smoker	8 (10.7)	34 (10.4)	0.892
Previous MI	2 (2.7)	16 (4.9)	0.545
History of CVA	4 (5.3)	11 (3.4)	0.495
Depression	17 (22.7)	74 (22.6)	>0.9
Anxiety	12 (16.2)	44 (13.4)	0.577

Precipitating/predisposing factors			
Emotional stress	36 (48.0)	179 (54.6)	0.31
Physical stress	21 (28.0)	89 (27.1)	0.89
Isometric >50 pounds	6 (28.6)	32 (36.0)	0.62
Connective tissue disease	6 (8.0)	13 (4.0)	0.13
Systemic inflammation	6 (8.0)	15 (4.6)	0.25
Active hormonal treatment	14 (18.7)	34 (10.4)	0.073
Postmenopausal	36/71 (50.7)	201/297 (67.7)	0.009
Peripartum	3/71 (4.2)	6/297 (2.0)	0.38
Fibromuscular dysplasia	35 (46.7)	210 (64.0)	0.008

Hospital presentation			
STEMI	38 (50.7)	65 (19.8)	<0.001
NSTEMI	37 (49.3)	263 (80.2)	<0.001
Peak trop I (median, IQR)	16.0 (3.2, 40.2)	6.1 (1.8, 15.1)	0.001
Peak high-sensitive trop T	990 (269, 5507)	572 (263, 1916)	0.399
VT/VF	10 (13.3)	28 (8.6)	0.272
EF (%)	50 (40, 50)	58 (50, 63)	<0.001
EF <50%	25 (40.3)	45 (18.1)	0.001
Hospital length of stay (days)	3 [3, 5]	3 [3, 4]	0.038

**Table 2:** Angiographic characteristics.

*n* (%), median (Q1, Q3)	PCI (*n* = 75)	No PCI (*n* = 328)	*P* value
TIMI flow			
0	19 (25.3)	54 (16.5)	0.10
1	18 (24.0)	40 (12.2)	0.016
2	13 (17.3)	45 (13.7)	0.46
3	25 (33.3)	189 (57.6)	<0.001
TIMI 0 or 1	37 (49.3)	94 (28.7)	0.001

SCAD type			
Type 1	28 (37.3)	84 (25.6)	0.046
Type 2	43 (57.3)	227 (69.2)	0.057
Type 2A	44.4%	45.5%	>0.9
Type 2B	55.6%	54.5%	>0.9
Type 3	4 (5.3)	17 (5.2)	>0.9
Lesion length (mm)	49 (30, 70.6)	39.3 (27.4, 56.2)	0.038
Lesion stenosis (%)	90.4 (70.5, 100)	77.6 (61.4, 100)	0.001
Stenosis severity 90–100%	39 (57.4)	108 (34.3)	0.001
Reference artery diameter (mm)	2.8 (2.4, 3.3)	2.2 (1.9, 2.6)	<0.001
LM involvement	4 (5.3)	0 (0)	0.001
LAD territory	44 (58.7)	154 (47.0)	0.074
LCX territory	19 (25.3)	112 (34.1)	0.17
RCA territory	14 (18.7)	91 (27.7)	0.11
LM or proximal segment	19 (25.3)	15 (4.6)	<0.001
Multivessel noncontiguous SCAD	10 (13.3)	30 (9.1)	0.28

**Table 3:** Rationale for PCI.

*n* (%)	PCI *n* = 75
Planned PCI as an initial strategy	60 (80.0)
PCI after failed med	11 (14.6)
PCI after failed thrombolysis	4 (5.3)
Indications for PCI	
Ongoing ischemia	28 (37.3)
Ongoing symptoms	19 (25.3)
VT or VF	7 (9.3)
Hemodynamic instability	3 (4.0)
LM dissection	4 (5.3)
Large artery ³3 mm	14 (18.7)
Proximal segments	19 (25.3)
Severe stenosis (90-100%)	25 (33.3)
TIMI 0 or 1 flow	37 (49.3)
Multivessel SCAD	4 (5.3)
Catheter-induced dissection	7 (9.3)
Other	6 (8.0)

**Table 4:** PCI procedural details and outcomes.

N (%), mean ± SD	Overall *N* = 75	Successful *N* = 26 (34.7)	Partial success *N* = 28 (37.3)	Unsuccessful *N* = 21 (28.0)
Procedures				
Wiring only	8 (10.7)	0 (0)	0 (0)	8 (38.1)
Balloon only	12 [16]	1 (3.8)	3 (10.7)	8 (38.1)
Cutting balloon	1 (1.3)	1 (3.8)	0 (0)	0 (0)
Stent	55 (73.3)	25 (96.2)	25 (89.3)	5 (23.8)
Unplanned stents	22 (40.0)	9 (36.0)	12 (48.0)	1 (20.0)
Mean number of stents	2.6 ± 1.8	2.6 ± 1.4	2.8 ± 2.2	1.4 ± 0.5
>3 stents used	11 [15]	5 [17]	6 (24)	0 (0)
Propagation of dissection	33 (44.0)	10 (38.5)	14 (50.0)	9 (42.9)
Residual dissection	44 (58.7)	0 (0)	24 (86.7)	20 (95.2)
Bailout emergency CABG	4 (5.3)	0 (0)	1 (3.6)	3 (14.3)
Stent thrombosis/closure	4 (5.3)	0 (0)	2 (7.1)	2 (9.5)

Final TIMI flow				
0	11 (14.7)	0 (0)	0 (0)	11 (52.4)
1	6 (8.0)	0 (0)	0 (0)	6 (28.6)
2	4 (5.3)	0 (0)	2 (7.1)	2 (9.5)
3	54 (72.0)	26 (100)	26 (92.9)	2 (9.5)

PCI effect on TIMI flow				
Improved	47 (62.7)	21 (80.8)	25 (89.3)	1 (4.8)
Unchanged	24 (32.0)	5 (19.2)	3 (10.7)	16 (76.2)
Worse	4 (5.3)	0 (0)	0 (0)	4 (19.0)

**Table 5:** In-hospital and postdischarge clinical adverse events.

N (%)	PCI (*n* = 75)	No PCI (*n* = 328)	*P* value
In-hospital events:			
Death	0 (0)	1 (0.3)	>0.9
MI	15 [17]	4 (1.2)	<0.001
Repeat revascularization	14 (18.7)	3 (0.9)	<0.001
CVA	3 (4.0)	2 (0.6)	0.047
In-hospital MACE	22 (29.3)	9 (2.8)	<0.001
			

Postdischarge events:			
Unstable angina hospitalization	10 (13.3)	19 (5.8)	0.043
Death	0 (0)	4 (1.2)	>0.9
Recurrent MI	15 [17]	59 (18.0)	0.74
Recurrent de novo SCAD	5 (6.7)	40 (12.2)	0.22
Repeat revascularization	11 (14.7)	10 (3.0)	<0.001
CVA	1 (1.3)	4 (1.2)	>0.9
Postdischarge MACE	18 (24.0)	65 (19.8)	0.43
			
Overall MACE	44 (58.7)	74 (22.6)	<0.001

## Data Availability

All data used in the study are part of the Canadian SCAD Registry and are subject to registry data storage protocols.
